# Pitfalls in compressed sensing reconstruction and how to avoid them

**DOI:** 10.1007/s10858-016-0068-3

**Published:** 2016-11-11

**Authors:** Alexandra Shchukina, Paweł Kasprzak, Rupashree Dass, Michał Nowakowski, Krzysztof Kazimierczuk

**Affiliations:** 1 Centre of New Technologies, University of Warsaw, Banacha 2C, 02-097 Warsaw, Poland; 2 Department of Mathematical Methods in Physics, Faculty of Physics, University of Warsaw, Pasteura 5, Warsaw, Poland; 3 Institute for Spectroscopy, Russian Academy of Sciences, Fizicheskaya 5, Troitsk, Moscow, Russia 108840

**Keywords:** Non-uniform sampling, CLEAN, Iterative soft thresholding, Iteratively re-weighted least squares, Matching pursuit, Low-rank

## Abstract

Multidimensional NMR can provide unmatched spectral resolution, which is crucial when dealing with samples of biological macromolecules. The resolution, however, comes at the high price of long experimental time. Non-uniform sampling (NUS) of the evolution time domain allows to suppress this limitation by sampling only a small fraction of the data, but requires sophisticated algorithms to reconstruct omitted data points. A significant group of such algorithms known as compressed sensing (CS) is based on the assumption of sparsity of a reconstructed spectrum. Several papers on the application of CS in multidimensional NMR have been published in the last years, and the developed methods have been implemented in most spectral processing software. However, the publications rarely show the cases when NUS reconstruction does not work perfectly or explain how to solve the problem. On the other hand, every-day users of NUS develop their rules-of-thumb, which help to set up the processing in an optimal way, but often without a deeper insight. In this paper, we discuss several sources of problems faced in CS reconstructions: low sampling level, missassumption of spectral sparsity, wrong stopping criterion and attempts to extrapolate the signal too much. As an appendix, we provide MATLAB codes of several CS algorithms used in NMR. We hope that this work will explain the mechanism of NUS reconstructions and help readers to set up acquisition and processing parameters. Also, we believe that it might be helpful for algorithm developers.

**Electronic supplementary material:**

The online version of this article (doi:10.1007/s10858-016-0068-3) contains supplementary material, which is available to authorized users.

## Introduction

The role of multidimensional NMR spectroscopy in the progress of biomolecular studies cannot be overestimated. The effectiveness of NMR is a fruit of decades-long efforts to improve spectrometer hardware, pulse sequences, sample preparation methods and, last but not least, signal processing techniques. Among the latter ones, non-uniform sampling (NUS) has become a standard solution to reduce data collection times.

To describe how NUS processing works, let us turn to linear algebra terms.

A usual signal processing task in NMR is to find the spectrum $\varvec{x}$ of a measured FID signal $\varvec{y}$ by solving the system of equations

1 $$\begin{aligned} \varvec{F} \varvec{x} = \varvec{y}, \end{aligned}$$

where $\varvec{F}$ is an inverse Fourier transform matrix. Conventional sampling fulfilling the Nyquist theorem (Nyquist 2002) corresponds to the full-rank square matrix $\varvec{F}$, and the number of unknowns matching the number of equations $|\varvec{x}|=|\varvec{y}|$. When sparse non-uniform sampling is employed, a shorter vector $\tilde{\varvec{y}}$ is acquired. Square inverse Fourier transform matrix $\varvec{F}$ in Eq. (1) is then replaced with a rectangular one, denoted $\tilde{\varvec{F}}$. The new system of equations, $\tilde{\varvec{F}} \varvec{x} = \tilde{\varvec{y}}$, becomes underdetermined ($|\varvec{x}|>|\tilde{\varvec{y}}|$) and can be solved only under certain additional assumptions. Numerous reconstruction methods exploiting NUS developed over the years used various kinds of assumptions, e.g. maximum entropy of $\varvec{x}$ (Hoch and Stern 2001), knowledge on empty regions in a spectrum (Matsuki et al. 2009) or models of a spectrum (Orekhov and Va 2011). Effective alternatives to NUS involved radial sampling approaches (Coggins et al. 2010) and non-FT methods for conventional sampling (Zhang and Brüschweiler 2004; Mandelshtam 2000).

Compressed sensing (CS), gaining popularity in NMR in recent years (Kazimierczuk and Orekhov 2011; Holland et al. 2011), is based on NUS and assumes that the spectrum is sparse, i.e., the number of significant points (*K*) is limited comparing to the size of full sampling/spectrum grid (*n*). Then, spectrum $\varvec{x}$ is found by $\ell _p$-norm ($0<p\le 1$) minimization:

2 $$\begin{aligned} \mathop {{{\mathrm{\arg \!\min }}}}\limits _{\varvec{x}\in \mathbb {C}^n}\ || \tilde{\varvec{F}} \varvec{x} - \tilde{\varvec{y}}||_{\ell _{2}}+\lambda || \varvec{x}||_{\ell _{p}} \end{aligned}$$

or

3 $$\mathop{\hbox{arg\,min}} \limits_{{\varvec{x}}\in {\mathbb{C}}^n} \Vert {\varvec{x}}\Vert_{\ell _p} \,\hbox{subject\,to}\, \Vert{\tilde{\varvec{F}}}{\varvec{x}} - {\tilde{\varvec{y}}}\Vert_{\ell_{2}} \le \epsilon.$$

It can be shown that for $x^*$ being a solution of (3) for some $\epsilon$, there exists certain $\lambda$ for which $x^*$ is also a solution of (2). Conversely, if $x^*$ solves (2) with a certain $\lambda$, then there exists $\epsilon$ such that $x^*$ solves (3) [see Theorem B.28 in (Foucart and Rauhut 2010)].

Cândes et al. (2006a) have shown that the $\ell _1$-norm constraint [$p=1$ in (3) and (2)] allows to find the sparsest spectrum that matches the experimental data $\tilde{\varvec{y}}$. This works equally well for $p<1$) (Foucart and Rauhut 2010, Proposition 3.2). A strictly sparse $\varvec{x}$ is reconstructed perfectly from the number of sampling points of the order of *K*log(*n*/*K*), where *n* is the full grid size, *K* denotes the number of non-zero points (Foucart and Rauhut 2010). The same applies to *K* highest points of approximately sparse spectra (Candès et al. 2006a). The latter statement is of crucial importance for the case of NMR. NMR spectra, though rarely being strictly sparse (a Lorentzian peak assymptotically approaches zero, but is never equal to it), are often approximately sparse: the number of points in a spectrum contributing to meaningful intensities is much less that the number of points contributing to noise. In this case, we can still successfully use CS approaches in NMR. The assumption of the sparsity of $\varvec{x}$ is more appropriate here if the imaginary part of a spectrum is zeroed by Virtual Echo (Mayzel et al. 2014), or simply the real part of a spectrum is taken as the second term of Eq. (2) (Stern and Hoch 2015).

Out of the algorithms proposed to solve Eq. (2), iterative soft thresholding (IST) (Kazimierczuk and Orekhov 2011; Hyberts et al. 2012b) with optimizations (Sun et al. 2015) and iteratively re-weighted least squares (IRLS) (Kazimierczuk and Orekhov 2011) are worth mentioning. Sparsity of a spectrum is also implicitly assumed in various modifications of the CLEAN algorithm (Barna et al. 1988; Kazimierczuk et al. 2007a; Coggins and Zhou 2008; Stanek and Koźmiński 2010a; Kazimierczuk and Kasprzak 2015). Recent algorithms adapted to exponentially decaying signals are also highly promising (Qu et al. 2015).

For strictly sparse signals, the result of $\ell _1$-norm minimization is known to be equivalent to the decomposition of the signal in an overcomplete basis (Chen et al. 2001). This resembles known singular value decomposition approach that was used for general spectral analysis prior to CS developments, as well as for sparsity-enhancing FID signal processing [e.g. de-noising (Fedrigo et al. 1996), solvent suppression (Zhu et al. 1997), signal extrapolation (Barkhuijsen et al. 1985)]. The invention of IST, which also solves the $\ell _1$-norm minimization problem (Stern et al. 2007), allowed to significantly decrease the computational complexity of the procedure. Moreover, not only strictly sparse cases, but also approximately sparse ones (NMR spectra among them) can often be effectively treated by this approach.

In the present paper we study conditions under which popular CS algorithms yield wrong reconstructions in NMR: either ignore peaks which should be present in a spectrum or produce false artificial peaks or peak splittings. So far, this topic has rarely been extensively discussed, especially in a comparative manner. Our goal is to show similarities between various CS methods, provide simple explanations of spectral distortions and ways to correct them. The reader is encouraged to verify the statements using the MATLAB codes provided as a Supplementary Material.

## Methods

This section presents definite CS reconstruction methods and experimental procedures used to verify them.

For the considerations below we will keep the following notations: $\varvec{y}$ is a measurement vector acquired with full sampling, $\tilde{\varvec{y}}$ is a measurement vector acquired with NUS (shorter than $\varvec{y}$, with certain data points skipped). Let us also introduce vector $\tilde{\varvec{y}}_0$ for NUS, of the same size as a fully sampled one, but with omitted data points set to zero.

As an introduction, we will explain the general principle of all CS reconstruction methods in terms of “artifact cleaning”. The FT of $\tilde{\varvec{y}}_0$, being a starting point of most of CS algorithms, differs from a perfect spectrum of full data $\varvec{y}$ by the presence of artifacts. The artifacts are the effect of the convolution of a perfect spectrum with FT of a sampling schedule. The latter FT is often called point spread function ($\text {PSF}$) (Kazimierczuk et al. 2007a; Hyberts et al. 2012a; Maciejewski et al. 2009). In case of NUS, the artifacts resemble noise, while in case of more regular sampling schedules [e.g. radial (Marion 2006), spiral (Kazimierczuk et al. 2006) or concentric (Coggins and Zhou 2007)] they take more regular form [see (Kazimierczuk et al. 2007a) for examples]. Strong artifact patterns originating from strong peaks can cover small resonances. Thus, it is usually desirable to clean them. Most popular CS methods iteratively deconvolve the spectrum from the artifact pattern.

These “NUS artifacts”, that are intuitively understandable for an NMR spectroscopist, have much in common with the mathematical concept of matrix coherence that underlies the CS theory. The coherence of an undersampled FT matrix $\tilde{\varvec{F}}$ is defined as a maximum among scalar products of all pairs of its columns $\varvec{f}$ [see also Definition 5.1 in (Foucart and Rauhut 2010)]:

4 $$\begin{aligned} \mu (\tilde{\varvec{F}}) :=\max _{i \in [n]} \max _{j \in [n] \backslash \{ i \} } |\langle \varvec{f}_i , \varvec{f}_j\rangle| \end{aligned}$$

where $[n]=\left\{ 1,2,\ldots ,n \right\}$. Let us note that the coherence of a matrix $\tilde{\varvec{F}}$ is equal to the highest artifact in $\text {PSF}(\omega )$, i.e., $\max _{\omega \ne 0}|\text {PSF}(\omega )|$.

A more general concept of $\ell _1$-coherence, or *s*-column-coherence (Definition 5.2 in Foucart and Rauhut 2010), predicts the worst-case artifact maximum resulting from the overlap of many PSFs with all possible relative positions. In other words, it gives the estimation for the highest artifact in a spectrum with *s* peaks at any positions:

5 $$\begin{aligned} \mu _s (\tilde{\varvec{F}}) :=\max _{i \in [n]} \max _{S \subseteq [n] \backslash \{ i \}, |S|\le s } \sum _{j \in S}| \langle \varvec{f}_i , \varvec{f}_j \rangle| \end{aligned}$$

The iterative deconvolution of the artifact pattern is more effective, if $\mu _s(\tilde{\varvec{F}})$ is small. For example, the theory guarantees, that one of the simplest CS algorithms, orthogonal matching pursuit (described below) reconstructs every vector $\varvec{x}$ with *s* non-zero elements after at most *s* iterations if:

6 $$\begin{aligned} \mu _s (\tilde{\varvec{F}})+ \mu _{s-1} (\tilde{\varvec{F}})< 1 \end{aligned}$$

[see Theorem 5.14 in (Foucart and Rauhut 2010)]. Experimental NMR signals, due to Lorentzian peak shapes, are not strictly sparse. In addition, they contain noise. Thus, the usability of $\mu _s(\tilde{\varvec{F}})$ is limited. However, the general rule, which binds the reconstruction performance with the artifact level in the direct FT of experimental data $\tilde{\varvec{y}}$, is true. It is generally recommended to have a look at the spectrum with artifacts before performing NUS reconstruction, to see how difficult it will be, and thus how credible the result might be.

### Compressed sensing algorithms

#### CLEAN and orthogonal matching pursuit (OMP)

A predecessor of *orthogonal matching pursuit* (OMP) algorithm has been known under the name of CLEAN in astronomy since 1974 (Högbom 1974). In 1988 (Barna et al. 1988), it was used in 2D NMR experiments for the first time, and was later improved (Kazimierczuk et al. 2007a; Coggins and Zhou 2008; Stanek and Koźmiński 2010a; Kazimierczuk and Kasprzak 2015). CLEAN belongs to a group of *greedy* CS methods, which means that it solves a global problem by making a locally optimal choice in each iteration. At first, we will describe its most basic version, known in CS literature as *matching pursuit* (Mallat 1993).

**Fig. 1:**
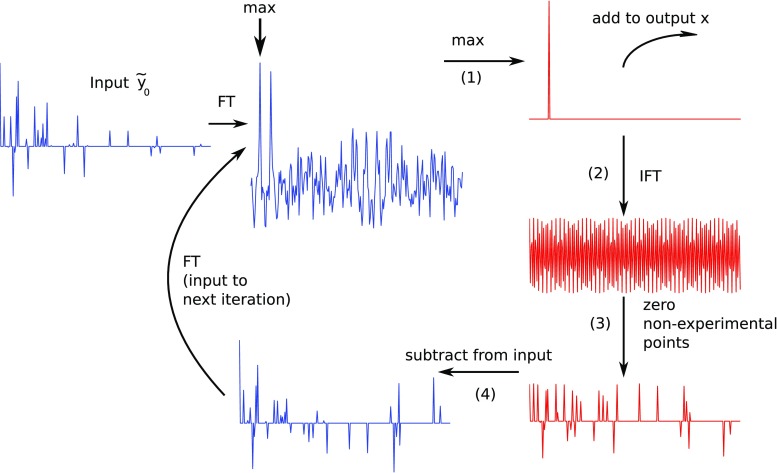
Overview of the CLEAN algorithm. Steps marked (*1*)–(*4*) described in the main text

The procedure of the algorithm is illustrated in Fig. 1. At the starting point (the first iteration), the sought vector $\varvec{x}$ is set to zero. At each iteration, the global maximum of the spectrum with artifacts (Fourier transform of $\tilde{\varvec{y}}_0$) is found. It is added to $\varvec{x}$ (step (1) in Fig. 1). Then, the inverse FT of the updated $\varvec{x}$ is taken (step 2). Data points corresponding to omitted measurements are set to zeros in this time-domain reconstruction (step 3). The result is subtracted from $\tilde{\varvec{y}}_0$ (step 4). The FT of this difference will give the updated spectrum with artifacts. At this point the next iteration begins. After a sufficient number of iterations, the output $\varvec{x}$, i.e., the reconstructed spectrum, will contain meaningful peaks, but not artifacts.

The difference between OMP and CLEAN is that in OMP the heights of *all* peaks acquired in $\varvec{x}$ are redefined at each iteration (which corresponds to the orthogonal projection of the signal $\tilde{\varvec{y}}$ onto a subspace spanned by the columns of $\tilde{\varvec{F}}$ corresponding to the positions of the peaks found so far, hence the name *orthogonal* matching pursuit), while in CLEAN the height of once-found peak is preserved throughout all iterations.

A more rigorous description of OMP is given in Algorithm 1. The set of indices that determine non-zero components of sparse vector $\varvec{x}$ is called the support of $\varvec{x}$. Here we will denote it as $I = {{\mathrm{supp}}}\, \varvec{x}$.

Two types of stopping criteria are commonly used in OMP and CLEAN. It is either the norm of the residual $\epsilon$ (the difference between initial $\tilde{\varvec{y}}$ and $\tilde{\varvec{F}}\varvec{x}$; $\epsilon$ should be set equal to the $\ell _2$-norm of noise in order to provide an optimal output) or the maximum number of iterations (equal to the maximum number of non-zero components in $\varvec{x}$). In Algorithm 1, both criteria are applied.

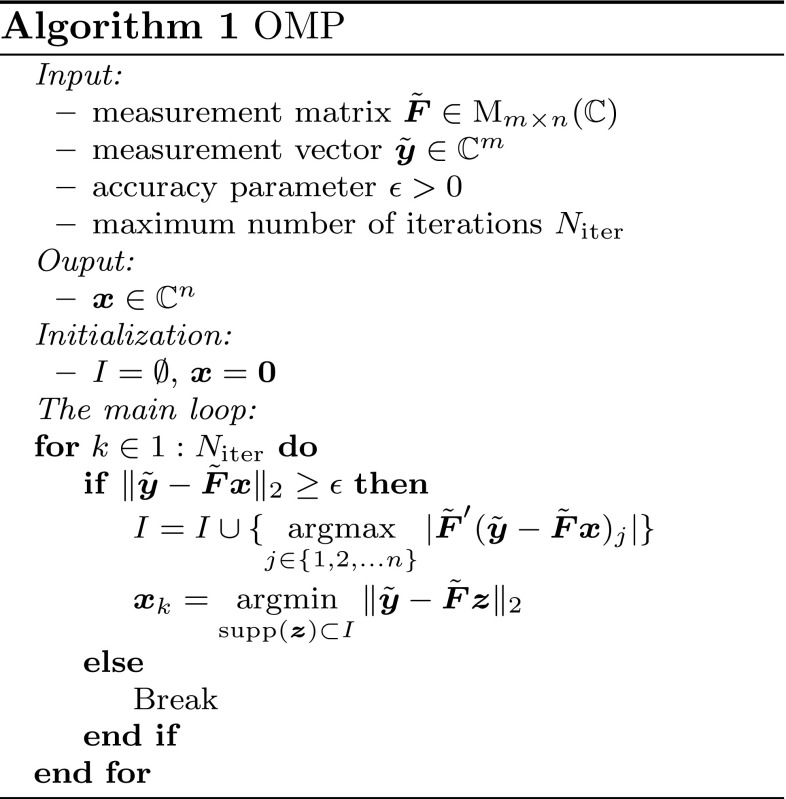



#### Iterative soft thresholding

As pointed out by Sun et al. (2015), in NMR literature there are two variants of the CS algorithm referred to as iterative soft thresholding (IST). One keeps the balance between the accordance with the data and sparsity, while the other enforces strict accordance with the measured data. The former was used in e.g. (Drori 2007; Hyberts et al. 2012b) and in recent versions of CS module in *mddnmr* software (Orekhov et al. 2004–2016), while the latter e.g. in Sun et al. (2015), Stern et al. (2007), and early works of our group (Kazimierczuk and Orekhov 2011). After Sun et al., we will call the first one IST-D and the latter IST-S.

**Fig. 2:**
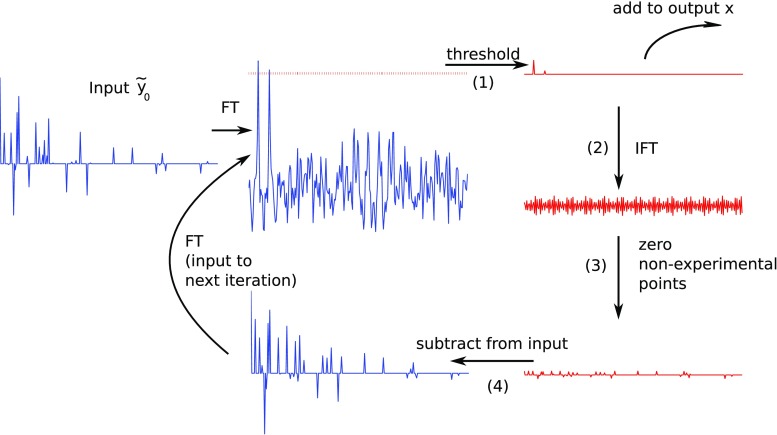
Overview of the IST-D algorithm solving the problem given by Eq. 3. Steps marked (*1*)–(*4*) described in the main text

IST-D is based on a similar idea as CLEAN, but, instead of selecting one highest point at each iteration, all points above a definite threshold are selected. As stated by Drori (2007), IST-D is equivalent to solving Eq. (3) with $p = 1$. The scheme for this algorithm is presented in Fig. 2 and in Algorithm 2. Steps (2)–(4) are the same as in CLEAN (compare Figs. 1, 2), and only step (1) differs. The output here consists of the sum of thresholded spectra from each iteration.

Similarly to CLEAN, the constraining parameter $\epsilon$ enters Algorithm 2 as a stopping criterion for the main loop. The smaller $\epsilon$ in Algorithm 2, the better the agreement of $\tilde{\varvec{F}} \varvec{x}$ with the measurement $\tilde{\varvec{y}}$, and the less sparse the output $\varvec{x}$ of Algorithm 2.

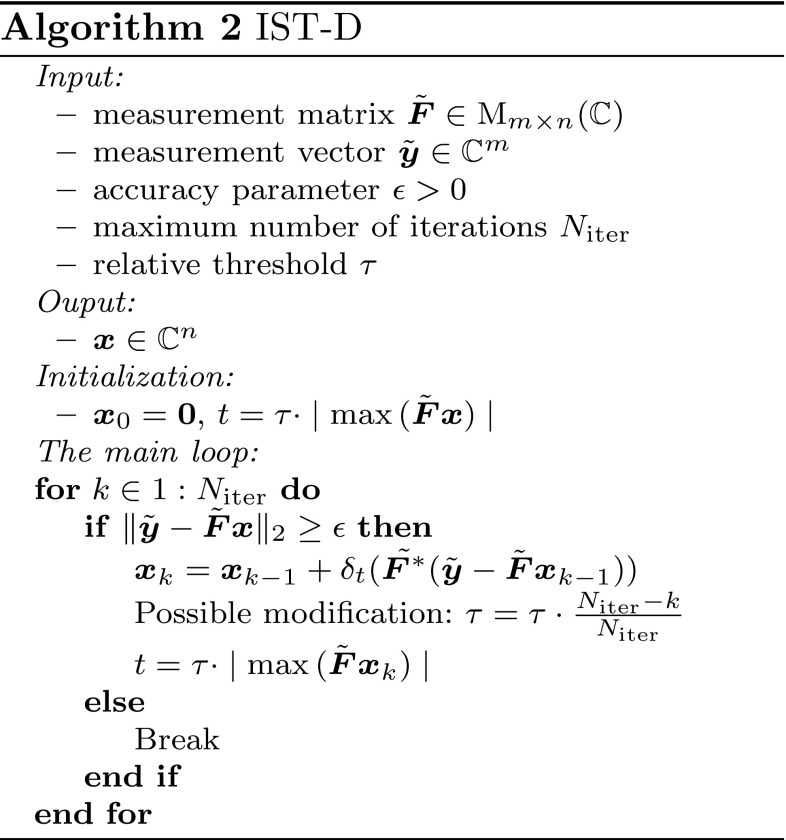



**Fig. 3:**
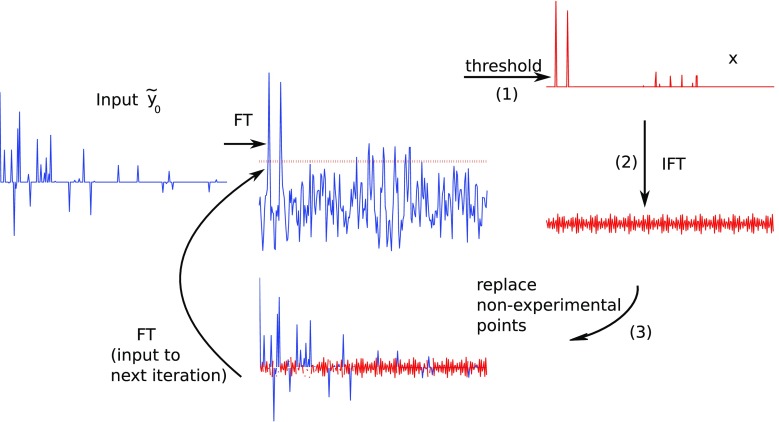
A scheme of IST-S algorithm solving problem given by Eq. (3). Steps marked (*1*)–(*3*) described in the main text

Another version of the algorithm, IST-S, is presented in Fig. 3. The difference between the two IST versions is in steps (3)–(4). After the inverse Fourier transform of updated $\varvec{x}$, measurement points omitted in NUS are not set to zeros, but their values are added to ${\tilde{\varvec{y}}_0}$ (initial zeros in ${\tilde{\varvec{y}}_0}$ are substituted with new values). Values of actually measured points are, on the contrary, kept constant throughout the procedure. The omitted measurements only are thus reconstructed in this version, whereas the measurements actually taken are not modified. The final iteration ends with the replacement step.

As shown by Stern et al. (2007), IST-S corresponds to solving Eq. (2) by conjugate gradient search. The two steps of the procedure, thresholding and replacement, correspond to the descent along the gradient of the second and the first term of the minimized function (Eq. 2). The assumption about the experimental error (noise) is implicitly contained in $\lambda$, a parameter of sparsity/data agreement balance introduced in Eq. 2.

The output of IST-S, according to Stern et al. (2007), should be the spectrum acquired at a certain number of iterations (enough for convergence) after the step (1), i.e. thresholding. It is also worth mentioning that then IST-S and IST-D converge to the same output. In practice, however, it is possible to perform the iteration to the end, i.e., carry out steps (2) and (3) as well, and take the FT of the result in step (3) as an output. In this case the exact data agreement with the measured data points of the FID is kept, and only the non-measured points are reconstructed. Then, the output of IST-D and IST-S differs.

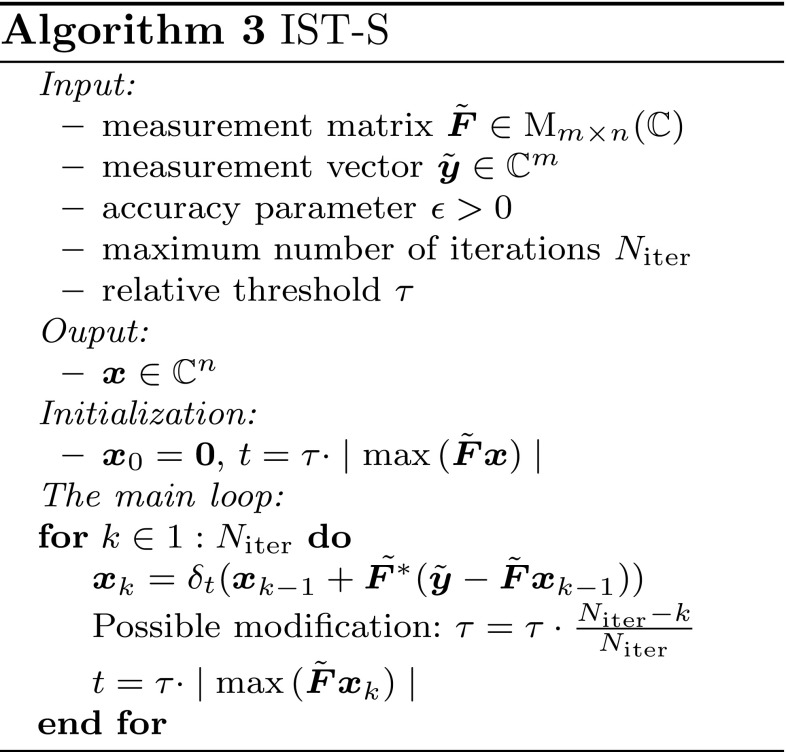



#### Iterative re-weighted least squares 

Iterative re-weighted least squares (IRLS) (Candès et al. 2008) reformulates the sparse reconstruction task (2) or (3) into regularized least-squares minimization problem. The Table 1 summarizes various least squares problems and their closed-form solutions. The standard least squares procedure is applied when the number of equations (i.e., the number of rows of the measurement matrix $\varvec{A}$ in the first row of Table 1) exceeds the number of unknowns. For NMR signal processing it never happens: the least-squares problem has infinitely many solutions, and each of them zeros the minimized function. However, the least squares problem can be uniquely solved in this case under some additional assumptions about the solution. One type of assumption (see the second row of Table 1) requires the vector satisfying the constraint $\tilde{\varvec{F}}\varvec{x} = \tilde{\varvec{y}}$ to have the smallest possible $\ell _2$-norm. Another common type of assumption, known as Tikhonov regularization, is particularly useful when the measurements are corrupted by noise. In this case the $\ell _2$-norm constraint is also employed. We present a closed form solution for this problem in the third row of Table 1 (note that it can be viewed as a modification of the solution in the second row of Table 1).

**Table 1:** Least squares problems: standard least-squares, Tikhonov regularization and re-weighted regularization

Name	Problem	Minimized function	Solution
Least squares	$\min _{\varvec{x}}||{\varvec{A}}\varvec{x}-\varvec{y}||^2$	$||{\varvec{A}}\varvec{x}-\varvec{y}||^2_{\ell _2}$	$\varvec{x} = ( {\varvec{A}}^{T} {\varvec{A}})^{-1}{\varvec{A}}^{T}\varvec{y}$
Least squares reg.	$${\left\{ \begin{array}{ll} \tilde{\varvec{F}}\varvec{x}= \tilde{\varvec{y}}\\ \min ||\varvec{x}||^2_{\ell _2} \end{array}\right. }$$	$||\varvec{x}||^2_{\ell _2}$	$\varvec{x} = \tilde{\varvec{F}}^{T}(\tilde{\varvec{F}}\tilde{\varvec{F}}^{T})^{-1}\tilde{\varvec{y}}$
Tikhonov reg.	$${\left\{ \begin{array}{ll} || \tilde{\varvec{F}}\varvec{x}- \tilde{\varvec{y}}||_{\ell _2}\le \epsilon \\ \min ||\varvec{x}||^2_{\ell _2} \end{array}\right. }$$	$||\tilde{\varvec{F}}\varvec{x}- \tilde{\varvec{y}}||^2_{\ell _2}+\lambda ||\varvec{x}||^2_{\ell _2}$	$\varvec{x} =\tilde{\varvec{F}}^T( \tilde{\varvec{F}} \tilde{\varvec{F}}^{T}+\lambda \mathbb {I})^{-1} \tilde{\varvec{y}}$
Re-weighted reg.	$${\left\{ \begin{array}{ll} ||\tilde{\varvec{F}}\varvec{x}- \tilde{\varvec{y}}||_{\ell _2}\le \epsilon \\ \min ||\varvec{W}\varvec{x}||^2_{\ell _2} \end{array}\right. }$$	$|| \tilde{\varvec{F}}\varvec{x}- \tilde{\varvec{y}}||^2_{\ell _2}+\lambda ||\varvec{W}\varvec{x}||^2_{\ell _2}$	$\varvec{x} = \varvec{W}^{-2} \tilde{\varvec{F}}^T( \tilde{\varvec{F}}\varvec{W}^{-2} \tilde{\varvec{F}}^T +\lambda \mathbb {I})^{-1}\tilde{\varvec{y}}$

The solution of Tikhonov regularization problem is not sparse: it can be shown that $\ell _p$-norms with $p>1$ do not yield sparse solutions [for simple example, see (Urbańczyk et al. 2016)]. However, the problem can be modified by introducing $\ell _p$-norm with arbitrary $0<\,p\le 1$, and thus ensuring that the spectrum is sparse. In this case, the problem does not have a closed-form solution, but has to be solved iteratively. At each iteration, the solution $\varvec{x}$ provides the weights $d_i = \left| x_i\right| ^{p-2}$ for the next iteration. They form the diagonal weight matrix $\varvec{W}^2 = \text {diag}(d_1, d_2 ,\ldots )$. Through this iterative process, the $\Vert \varvec{x}\Vert ^p_{\ell _p}$ norm is approximated by the weighted $\ell _2$-norm $\Vert \varvec{W} \varvec{x}\Vert ^2_2$. After sufficiently many iterations, we get $\varvec{x}$ which approximates the solution of (2).

The IRLS implementation uses two positive parameters denoted by $\varepsilon$ and $\lambda$. The first parameter $\varepsilon$ takes into account the fact that some coordinates of $\varvec{x}$ can be equal to zero (which makes the weights $w_i = \left| x_i\right| ^{p-2}$ ill-defined). The second parameter $\lambda$ balances the agreement of the solution $\varvec{x}$ to the measurement data and the sparseness of $\varvec{x}$. A remarkable modification of IRLS uses a third parameter $\delta$ corresponding to a small decrease of *p* in each iteration (Yagle 2009). After $k\approx \frac{1}{\delta }$ iterations, we get an approximate solution $\varvec{x}$ of $\ell _0$-optimization problem. The implementation of the $\delta$-modified version of IRLS is described in Algorithm 4.

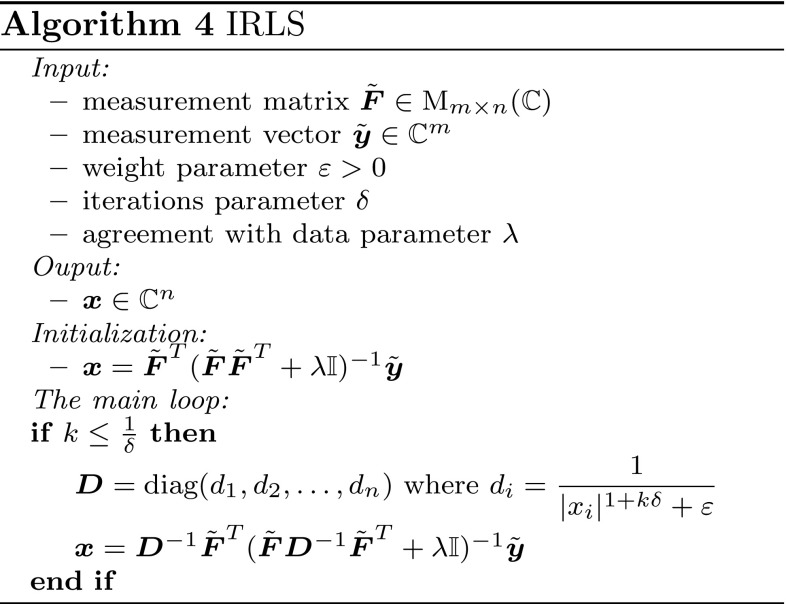



#### Low-rank reconstruction

In the general problem of compressed sensing, the assumption that the sought-for spectrum is sparse can be expressed in various ways. In the algorithms described above, sparsity implied that most of the spectrum components are close to zero. Now, we will present a method exploiting another approach to sparsity: as few peaks in the spectrum as possible. The method is referred to as low-rank reconstruction and was introduced into NMR by Qu et al. (2015). Within the framework of this approach, a spectrum consisting of one, possibly broad, Lorentzian peak and thus having many non-zero components will theoretically be considered strictly sparse, unlike in previous methods. One way to quantify the number of peaks in a spectrum is to calculate the nuclear norm of a Hankel matrix made up of the FID signal. Hankel matrix is a matrix of the following form:

7 $$\begin{aligned} \varvec{H}(\varvec{f}) = \varvec{R} \varvec{f} = \begin{bmatrix} f_{1}&f_{2}&f_{3}&\dots&f_{Q} \\ f_{2}&f_{3}&f_{4}&\dots&f_{Q + 1} \\ \vdots&\vdots&\vdots&\ddots&\vdots \\ f_{n - Q}&f_{n - Q + 1}&f_{n - Q + 2}&\dots&f_{n - 1} \\ f_{n - Q + 1}&f_{n - Q + 2}&f_{n - Q + 3}&\dots&f_{n} \end{bmatrix}, \end{aligned}$$

where $f_i$ is the *i*-th measurement point of the fully sampled FID (*i* runs from 1 to *n*). The nuclear norm, denoted as $||...||_*$, is the sum of singular values of a matrix. Thus, the problem of sparse reconstruction can be formulated as:

8 $$\begin{aligned} \underset{x}{\text {min}} (\Vert \varvec{R} \varvec{x}\Vert _* + \alpha \Vert \varvec{y}\ - \varvec{U} \varvec{x} \Vert _2^2). \end{aligned}$$

$\varvec{R}$ here is the operator that rearranges $\varvec{x}$ into a Hankel matrix. The number *Q* of its rows should be from 2 to $n - 1$. In practice, *Q* should be chosen so that it is bigger than the expected number of meaningful peaks in the spectrum.

Singular values are an analog of eigenvalues for non-square matrices: they are square roots of eigenvalues of $\varvec{H}^T \varvec{H}$ (and equally of $\varvec{H} \varvec{H}^T$). It can be shown that the number of non-zero singular values of matrix $\varvec{H}$ is equal to the number of linearly independent rows of this matrix. The latter, in its turn, is equal to the number of decaying oscillations of definite frequencies in the FID (due to the autoregressive properties of the FID also exploited in linear prediction methods)—in other words, to the number of Lorentzian peaks in spectrum of $\varvec{x}$. Thus, when the sum of singular values of the FID Hankel matrix is minimized, the number of peaks in the spectrum is minimized as well [see (Qu et al. 2015)].

$\varvec{U}$ in (8) is an operator that selects the points actually sampled in the experiment from the full FID vector $\varvec{x}$. Notably, the reconstruction procedure is performed exclusively in the time domain. Thus, the output of the algorithm will not be the reconstructed spectrum, but the reconstructed FID.

$\alpha$ in (8) is the data agreement parameter which defines the balance between the data agreement and the sparsity of $\varvec{x}$.

For a general outline of the low-rank matrix completion method it is enough to state the following steps: (1) the initial solution is constructed as $\varvec{x} = \varvec{U}^T \varvec{y}$; (2) Hankel matrix of the form (7) is constructed out of this solution; (3) the nuclear norm (the sum of singular values) of this matrix is minimized, balanced with a data agreement term according to (8). This final step is realized by thresholding the singular values of this matrix. That is, all singular values lower than some definite threshold are set to zero (soft thresholding).

For a more detailed and formal description, it should be mentioned how the third step is exactly realized. It is done by:

- transforming the unconstrained minimization into the constrained one with the help of a new variable $\varvec{Z}$, and adding Lagrangian multipliers $\varvec{D}$:
  9 $$\begin{aligned} \underset{D}{\text {max}}\quad \underset{x, Z}{\text {min}} (\Vert \varvec{Z}\Vert _* + \alpha \Vert \varvec{y}\ - \varvec{U} \varvec{x} \Vert _2^2 + \langle \varvec{D}, \varvec{R} \varvec{x} - \varvec{Z} \rangle ). \end{aligned}$$
  Here $\langle ... \quad , \quad ... \rangle$ is the real part of the inner product of two matrices;
- using the augmented Lagrangian with a parameter $\beta > 0$:
  10 $$\begin{aligned} \underset{D}{\text {max}} \quad \underset{x, Z}{\text {min}} (\Vert \varvec{Z}\Vert _* + \alpha \Vert \varvec{y}\ - \varvec{U} \varvec{x} \Vert _2^2 + \langle \varvec{D}, \varvec{R} \varvec{x} - \varvec{Z} \rangle + \beta \Vert \varvec{Rx} - \varvec{Z} \Vert ^2_F), \end{aligned}$$
  where $\Vert ...\Vert _F$ is the Frobenius norm of a matrix: $\Vert \varvec{M}\Vert _F = \sqrt{\sum \limits _{i=1}^m \sum \limits _{j=1}^n |M_{ij}|^2}$;
- solving (10) with the gradient ascent method with respect to $\varvec{D}$, with a step size $\tau$.

During the last step, the necessity to threshold the singular values of $\varvec{H}$ arises. The exact solution to the problem as described in Qu et al. (2015) is presented in Algorithm 5.

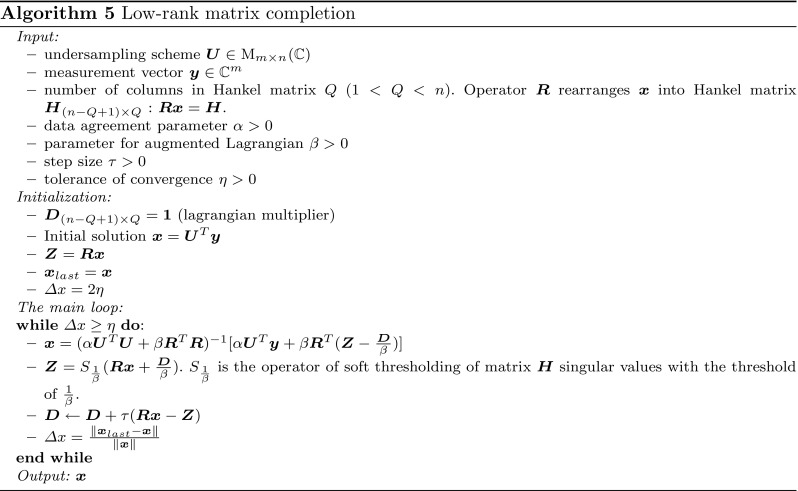



### Algorithms performance: theoretical outlook

The real efficiency of the particular NUS reconstruction program is as much dependent on the principles of the core algorithm as on other factors, such as automatic setting or optimal hard-coding of parameters, etc. Nevertheless, some summary of the theoretical facts about the aforementioned algorithms can be given:

- The CLEAN algorithm is the fastest, but fails in case of spectra with a high dynamic range of peak intensities, unless modified (Stanek and Koźmiński 2010a; Coggins and Zhou 2007). Its efficiency relies strongly on the validity of the stopping criterion.
- IST is somewhat slower but effective even for NOESY spectra. Based on FFT, it does not have high numerical requirements and can converge very rapidly if optimized (Sun et al. 2015).
- IRLS has higher numerical requirements than IST, as it involves matrix inversion, which has to be stored in the memory. It is typically faster than IST only for small 2D datasets, with numerical requirements rising with the number of time-domain points to power 3. However, it can provide better reconstructions at low sampling levels (Kazimierczuk and Orekhov 2012), which is in line with observations from other fields (Chartrand 2007).
- The low-rank method is theoretically best adapted to NMR spectra, as the FID signal becomes strictly sparse when put into a Hankel matrix. So far, however, the possible advantages of the low-rank method over classical CS approaches have been shown only on simulations (Qu et al. 2015). Current implementations of the low-rank method are limited to 2D spectra and are slower than IRLS.

### Experiments

We have applied the CS algorithms described above to various kinds of 2D and 3D spectra. In particular, we have been interested in practical aspects of the reconstruction: the minimum level of sampling sparseness providing good quality spectra and its dependence on the size of the full sampling grid; the consequences of missetting of parameters (sparsity constraint) and attempts to extrapolate the signal using CS methods.

Sampling schedules used to provide NUS data below are constructed by selecting a given number of indices *m* out of the full grid *n* with uniform probability.

#### Small molecule spectra

Three samples were prepared. The first sample was prepared by mixing 10.8 mg of glucose in 600 μl $\hbox {D}_{2}\hbox {O}$. The second sample contained 20.52 mg of maltose in addition to 10.8 mg of glucose in 600 μl $\hbox {D}_{2}\hbox {O}$. Similarly, the third sample contained 9mg of xylose in addition to 20.52 mg of maltose and 10.8 mg of glucose in 600 μl $\hbox {D}_{2}\hbox {O}$. Thus, the concentration of all compounds was 100 mM.

The experiments were performed on an Agilent 600 MHz DDR2 NMR spectrometer equipped with a triple-resonance HCN probe. All the measurements were performed at 298 K. The experiments were performed with conventional ${}^{13}\hbox {C}$ HSQC pulse sequence with no multiplicity editing. Hard pulses of 8 μs for ${}^{1}{\mathrm{H}}$ and 17.1 μ for ${}^{13}{\mathrm{C}}$ were used. The spectral widths were 30,166 Hz (${}^{13}{\mathrm{C}}$) and 9,615 Hz (${}^{1}{\mathrm{H}}$). An interscan delay of 2 s was used. The sampling was performed with 512 points with two scans per point. The NUS datasets were created by taking the subsets of the data from the full dataset. For tests of sampling sparseness, the IRLS algorithm with 20 iterations was used for the reconstruction at sampling levels from 16 to 512 points (Kazimierczuk and Orekhov 2011). IRLS was chosen taking into account the considerations given above in the “Algorithms performance—theoretical outlook”.

Additionally, a NOESY spectrum of the aforementioned mixture of glucose, maltose and xylose was measured using a conventional pulse sequence and a full sampling grid of 512 points in the indirect dimension. The spectral widths were set to 9615.384 Hz in both dimensions. Four points per scan were used with an interscan delay of 2 s. The mixing time was kept at 0.2 s.

#### Protein spectra

Protein experiments were performed on double-labeled SH3 domain of alpha spectrin protein from chicken brain (1mM protein sample in 10/90 % $\hbox {D}_{2}\hbox {O}/\hbox {H2O}$, 10 mM sodium citrate, 0.02 % NaN_3_, pH 3.5 obtained from Giotto Biotech). Measurements were performed on a Varian 700 MHz DDR2 spectrometer equipped with a triple-resonance room-temperature HCN probe at 298 K.

For the signal extrapolation test, the ${}^{15}{\mathrm{N}}$ HSQC pulse sequence was used (Kay et al. 1992) with 1 s recycle delay, 8 scans and 2056 points in the indirectly measured dimension.

For the test of minimum sampling sparseness, the HSQC experiment was repeated with 128 sampling points and 4 scans. The 3D HNCO experiment was run on the same sample with 128 points in ${}^{15}{\mathrm{N}}$ dimension and 64 points in CO dimension. The IRLS algorithm with 20 iterations was used for the reconstruction at sampling levels differing by 5 points between 15 and 125 NUS points for the HSQC and 15 to 900 NUS points for the HNCO. The results were averaged over 20 different sampling schedules for each sampling level.

## Results and discussion

### Sampling sparseness

One of the basic theorems of compressed sensing binds the number of sampling points *m* needed for a good reconstruction with a number of significant points in a spectrum, *K* [see equation 1.3 in (Foucart and Rauhut 2010)]. Namely, *m* should be in the order of $K\log (n/K)$, where *n* is the size of a full grid. In fact, the relation has a probabilistic form, and it is only *a chance* of a good reconstruction that grows with the number of samples.

On the other hand, in the literature on fast NMR methods as well as in many software packages the term “sampling sparseness” is often used to denote a percentage of *n* to be measured. Many authors state that certain minimum percentage of *n* is typically required for the reconstruction, suggesting the relation in the form of $m=a \cdot n$ (Sidebottom 2016; Le Guennec et al. 2015; Foroozandeh and Jeannerat 2015; Hyberts et al. 2014). Such a relation is in obvious contradiction to $K\log (n/K)$. It is true that we do not know the number of significant points *K* beforehand; thus, we do not have the possibility to apply expression $m \sim K\log (n/K)$ directly before NUS measurements to establish the number of points *m* that should be measured. This is the reason why some rules of thumb were developed, including those of the percentage formulation. However, one should be careful with them and bear in mind that they do not reflect the mathematical basis of CS. This point was also raised by other authors (Hyberts et al. 2014).

**Fig. 4:**
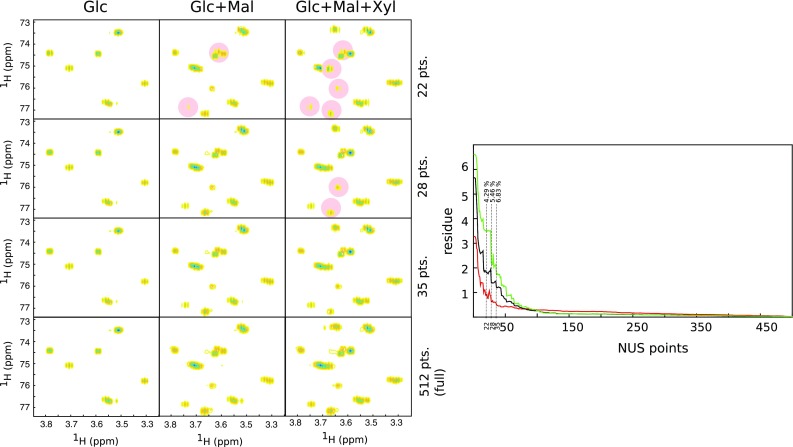
Results of experiment checking *K*log(*n*/*K*) relation. *Left panel* sugar region of spectra 2D ${}^{13}{\mathrm{C}}$ HSQC of various mixtures of glucose, maltose and xylose at various sampling levels. *Right panel* the fidelity of a reconstruction (residue) vs. the number of NUS points used for glucose (*red*), glucose+maltose (*black*) and glucose + maltose + xylose (*green*)

Figure 4a shows the spectra of three samples of carbohydrates: one-, two- and three-component mixtures at various levels of sampling. The data were acquired under the same experimental conditions, the only thing that differs is a number of peaks and thus a number of “significant” spectral points *K*. As expected from CS theory, with growing *K*, the growing levels of sampling are required to reconstruct the spectrum. All peaks in one-component (glucose only) spectrum seem to be reconstructed from even 22 points, while the three-component spectrum requires ca. 35 points.

It is interesting to see what happens when the number of sampling points is too low to reconstruct all peaks properly. According to CS theory, even at low *m* the highest *K* points of the spectrum should still be recovered well [see Theorem 1.2 in (Candès et al. 2006b)], while others are suppressed. This can be seen from spectra in Fig. 4. Also, a glance at residuals plot in Fig. 4 shows that the behavior of the algorithm for NUS NMR spectra is in line with the theory. Initially, the curve declines rapidly—this is when peaks are being reconstructed. For higher sampling levels, low spectral points (noise) are also being recovered, which corresponds to the plateau region. The plot is smooth and goes down monotonically, so it would be possible to implement the concept of Targeted Acquisition that was used before for the MDD method (Jaravine and Orekhov 2006); it is based on the on-the-fly processing of the data during an experiment and stopping it when the number of spectral peaks stabilizes.

**Fig. 5:**
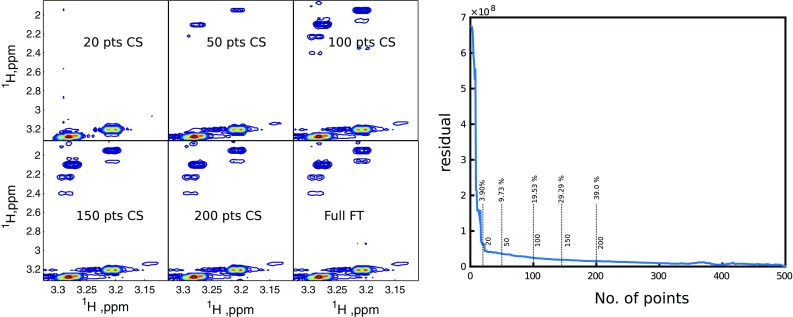
Results of experiment checking *K*log(*n*/*K*) relation for a NOESY spectrum with high dynamic range of signal intensities. *Left panel* part of 2D NOESY spectrum of a mixture of glucose, maltose and xylose at various sampling levels. *Right panel* the fidelity of a reconstruction (residue) vs. the number of NUS points

Keeping in mind the impressive results from 2D HSQC spectra that are well reconstructed from a very small fraction of the data, let us now turn the results of a similar reconstruction for NOESY spectrum of the three-component sample shown in Fig. 5. Although the plot of the residuals looks similar for HSQC spectra, much more NUS points are required to reconstruct small cross-peaks. This is because the diagonal peak is so much stronger than off-diagonal cross-peaks and thus has bigger contribution to the sparsity term in the penalty function. The algorithm starts to reconstruct small peaks only if *m* is high enough to well reconstruct more significant non-zero points of the spectrum, which contribute mostly to the diagonal peak.

It should also be borne in mind that many of NUS reconstruction software packages reconstruct indirect spectral dimensions separately for each point of the direct dimension (after FT of the direct dimension signal). This means that the condition *K*log(*n*/*K*) has to be considered separately for each “column” of 2D spectral matrices from Fig. 4. It may happen that for some of them the number of sampling points is sufficient to reconstruct all the peaks, while for others it is not. As a result, peaks may be missing or narrowed in both dimensions.

**Fig. 6:**
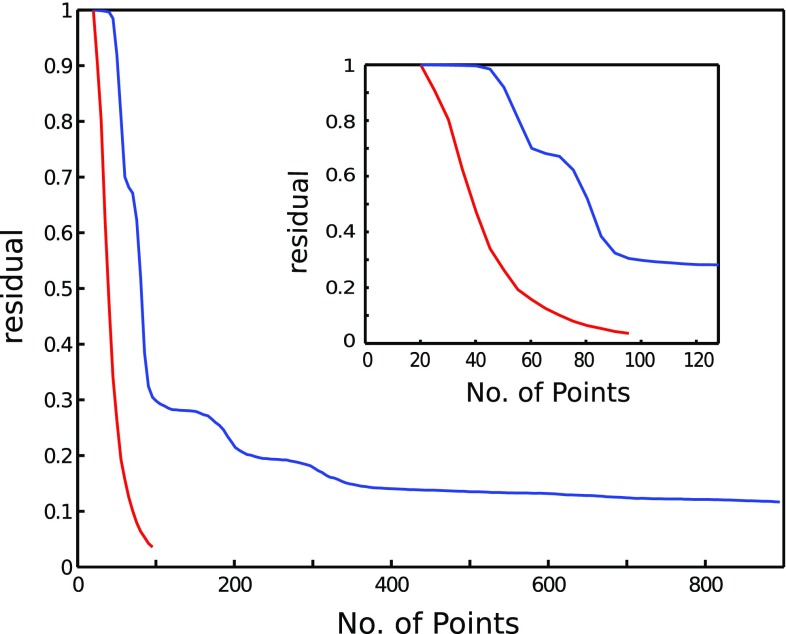
Results of experiment checking *K*log(*n*/*K*) relation for growing dimensionality. A narrow region of direct dimension (8.885–8.845 ppm) was taken for the reconstruction. The fidelity of a reconstruction (residue) vs. the number of NUS points is plotted for 2D ${}^{15} {\mathrm{N}}$ HSQC (*blue*) and 3D HNCO (*red*) spectra. The residue was calculated and averaged for 20 different sampling schedules for each sampling level

Another consequence of $m \propto Klog(n/K)$ relation can be seen if we consider growing spectral dimensionality without changing *K*, e.g., acquiring 2D HSQC and 3D HNCO spectrum of the same sample (as shown in Fig. 6). The number of points contributing to peaks scarcely changes—only the size of the full grid *n* differs. However, the required number of sampling points depends on log(*n*/*K*), so the difference is rather small. This again shows that referring to relative sampling sparseness expressed in a percentage of *n* can really be misleading.

It is interesting to observe how four peaks in HNCO spectrum are reconstructed consecutively for growing sampling level, which corresponds to “stairs” on the curve in Fig. 6.

To conclude this part, setting the sampling level in the experiment, one should rather compare the absolute number of sampling points to a number of highest spectral points to be reconstructed than to the size of the full sampling grid. For 2D spectra with large grids and low number of peaks (e.g. broad-band ${}^{13}{\mathrm{C}}$ HSQC) or high dimensional spectra (3D+), this may lead to huge time savings. For spectra with many peaks differing significantly in the intensity (like NOESY), the gain is less. In other words, if a spectrum is highly compressible, then the significant reduction of experimental time is possible.

It should also be mentioned that, for an arbitrary spectrum, the proportion between *m* and *K*log(*n*/*K*) does not depend on a particular sampling schedule, but on the type of sampling ((Foucart and Rauhut 2010), Theorem 9.2 on page 273). Several approaches to provide a better sampling type for NMR signals have been proposed recently (Eddy et al. 2012; Hyberts et al. 2010; Kazimierczuk et al. 2008, 2007b). To simplify the discussions in this paper, we use NUS with uniform density, as described in section “Experiments”.

### Algorithm parameters

All of the CS algorithms described above require certain parameters to be set by the user. In particular, the balance between the sparsity of the result and the accordance with the measured data ($\lambda$) and parameters associated with the thermal noise level (stopping criteria) are worth discussing.

Most often, such parameters are set automatically according to definite assumptions. Let us, however, investigate here the consequences of their missetting.

**Fig. 7:**
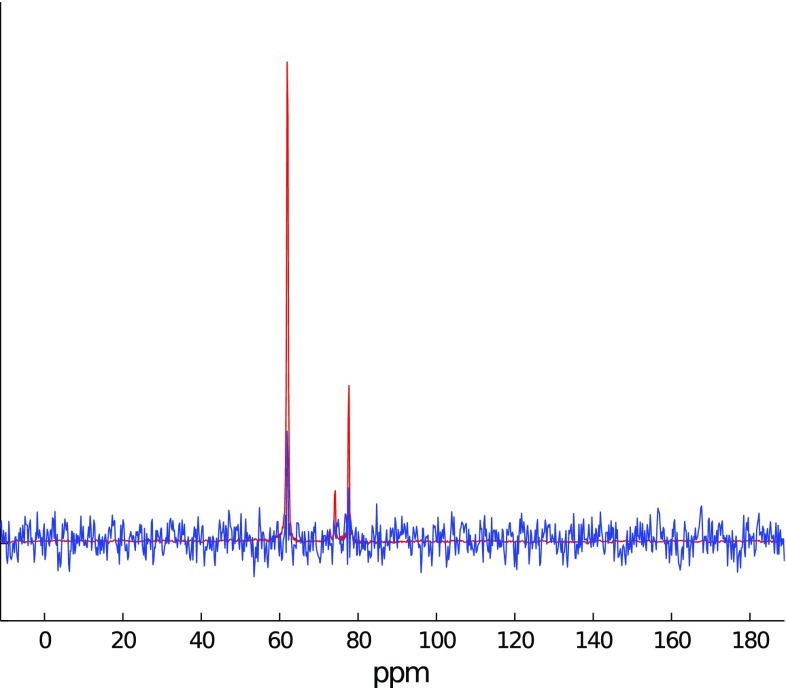
A row from the glucose-maltose 2D spectrum. *Red*—512 measurement points. *Blue*—spectrum with artifacts from NUS data (64 NUS points zero-filled to give the spectrum of the same length)

For this aim, we have taken the spectrum of the glucose and maltose mixture described above. We have selected a definite row (512 measurement points long) of this 2D spectrum and undersampled it to 64 NUS points (see Fig. 7). Then, we applied different algorithms (OMP, 2 versions of IST, IRLS and Low Rank reconstruction) varying their input parameters. The results are presented in the figures below.

Here we present qualitative results only. The quantitativeness of CS reconstruction, which is particularly important e.g. in relaxation studies, has recently been extensively studied (Stetz and Wand 2016; Linnet and Teilum 2016). Generally, two factors may disturb the relative intensities: too low sampling level as compared to the number of significant points and too high sparsity constraint ($\lambda$) . Both may cause the suppression of lower peaks. As predicted by the theory, the reconstruction fidelity at low sampling levels is better when IRLS is applied (Linnet and Teilum 2016). Even this approach, however, is very ineffective when compared to model-based MDD method, which treats serial 2D relaxation data as one object (Linnet and Teilum 2016). Now, let us turn to qualitative results of the algorithms described above.

**Fig. 8:**
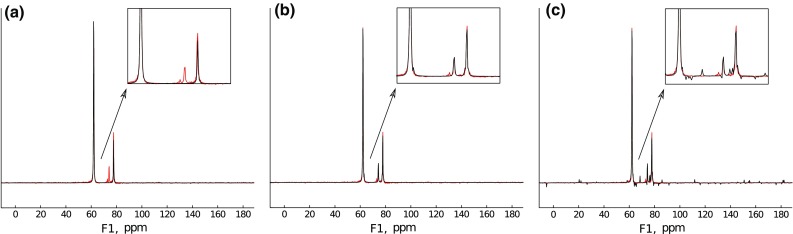
Results of processing for OMP algorithm: **a** high stopping criterion ($\times7$ average noise). **b** Optimal stopping criterion ($\times3$ average noise). **c** Low stopping criterion ($0.5\times$ average noise)

OMP results are presented in Fig. 8. The output spectrum depends here solely on the stopping criterion. The noise level can be used for this aim: when the algorithm starts producing peaks within the noise level, it should be stopped. Figure 8 presents the cases of overestimated noise level, optimally chosen one and underestimated one (stopping the algorithm too early, optimally and too late, accordingly).

It is worth mentioning that normally OMP does not provide smooth peaks but splits a Lorentzian peak into separate narrow neighbouring peaks. Here, exponential weighting is applied to the output, which hampers this effect.

**Fig. 9:**
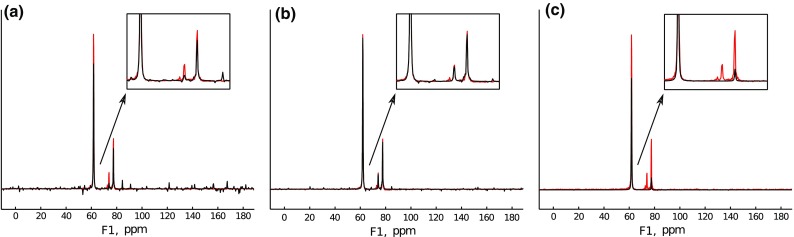
Results of processing for IST-D algorithm: **a** Low threshold = 0.25. **b** Optimal threshold = 0.9. **c** High threshold = 0.998

Parameters required for IST are: (1) threshold, and (2) stopping criterion. We took various thresholds allowing the algorithm run till convergence (optimal stopping criterion). The cases of too low threshold, optimal one and too high one are plotted in Fig. 9. Here, IST-D algorithm is used. With too low threshold, the reconstructed spectrum, clearly, has unsuppressed artifacts; with too high threshold it fails to reconstruct all the peaks. The threshold here corresponds to the assumed sparsity level.

For IST-S, which keeps the strict accordance with the measured data, the optimal result is practically the same as for IST-D. With too low initial threshold, it also has similar artifacts as in Fig. 9a. However, it is hardly possible to force IST-S to neglect peaks, as the threshold here has to be decreased from iteration to iteration.

**Fig. 10:**
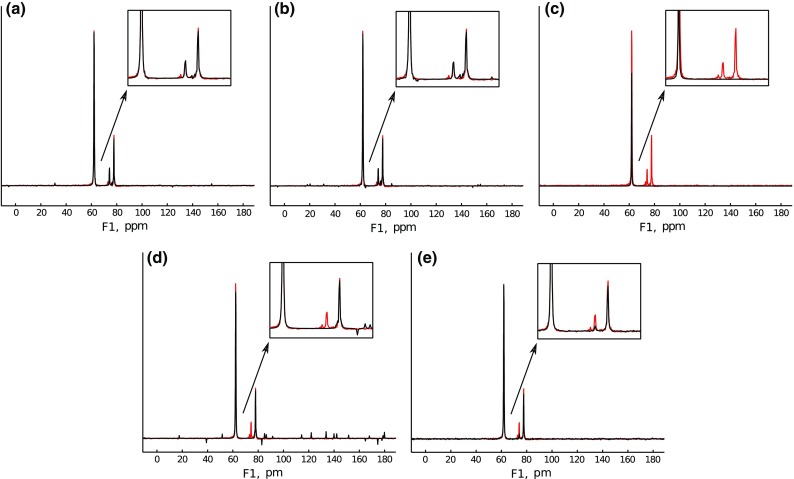
Results of processing for IRLS algorithm: **a** Optimal $\epsilon = 10^4$ and $\lambda$ = 500. **b** Optimal $\epsilon = 10^4$, low $\lambda = 10^{-5}$. **c** Optimal $\epsilon = 10^4$, high $\lambda = 10^5$. **d** Low $\epsilon = 10$, optimal $\lambda = 500$. **e** High $\epsilon = 10^5$, low $\lambda = 10^{-5}$

For IRLS, the parameters are: (1) the sparsity “weight” $\lambda$, which sets the balance between the sparsity constraint and the data agreement; (2) the regularization parameter $\varepsilon$; (3) the norm used in Eq. (2). Here, we kept the norm equal to 0.5 and changed $\lambda$ and $\varepsilon$.

As can be seen from Fig. 10a–c, with optimal $\varepsilon$, both optimal and low $\lambda$ give good reconstruction. With too high $\lambda$, peaks are neglected, as Fig.10c shows (this happens for any value of $\varepsilon$). With too low $\varepsilon$, even optimal $\lambda$ leads improper reconstruction, as in Fig. 10d. With too high $\varepsilon$, even low $\lambda$ (meaning low sparsity) leads to peak neglection (Fig. 10e).

**Fig. 11:**
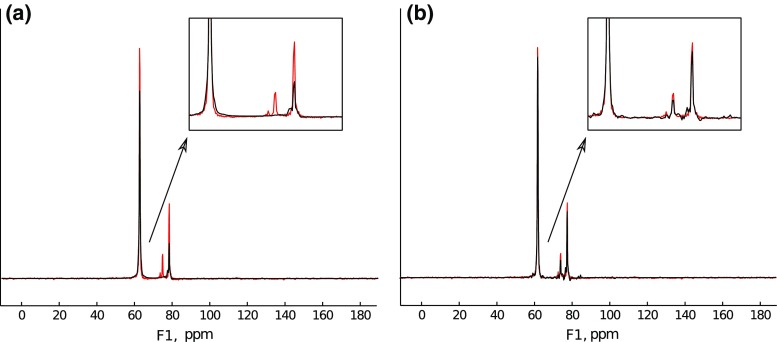
Results of processing for low-rank algorithm: **a** Low $\alpha$ = 20. **b** Optimal $\alpha$ = 1000

For low rank reconstruction, the idea of balancing the sparsity and data agreement is similar, but, instead of using the factor $\lambda$ giving the “weight” of the sparsity term, here factor $\alpha$ for the data agreement term is used. In Fig. 11, two cases are shown: too low $\alpha$ and the optimal one. When the data agreement term is underestimated, the algorithm, despite neglecting some of the peaks, produces a broader peak instead of two (or more) neighboring ones. Here, it tried to broaden the peak at 62 ppm to compensate for neighbouring noise peaks.

### Signal extrapolation

To study the effectiveness of signal “extrapolation” using CS algorithms, the FID from the protein ${}^{15} {\mathrm{N}}$ HSQC experiment described above has been used. A definite column of the 2D spectrum was selected (2056 measurement points long). Then, the FID was truncated to various lengths, and missing points at the end were reconstructed with IST-D.

**Fig. 12:**
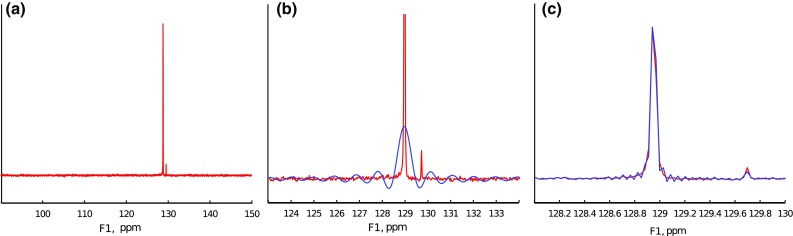
A cross-section through indirect dimension of 2D ${}^{15} {\mathrm{N}}$ HSQC spectrum (**a**) and spectra of signal truncated to 64 and 1024 points (*blue* in panels (**b**) and (**c**), accordingly). **c** is zoomed-in to visualize “sinc” wiggles

The initial spectrum, as well as the spectra of the FID truncated to 64 and 1024 points out of 2056 (magnified), are presented in Fig. 12.

We have applied IST-D with various levels of sparsity $\lambda$ (i.e., threshold values) to both cases.

**Fig. 13:**
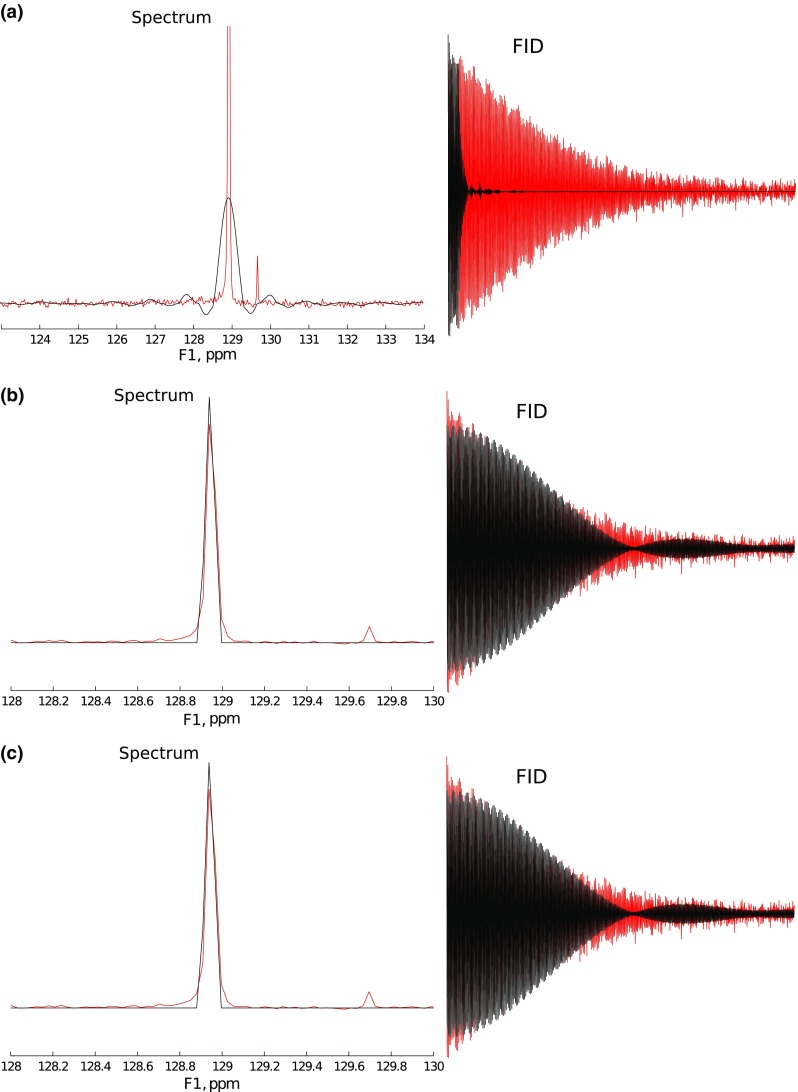
Extrapolation of 64 points signal with IST with various thresholds: **a** Low threshold (0.2). **b** Optimal threshold (0.99). **c** High threshold (0.999)

The results of reconstruction for the extreme truncation (64 points) are given in Fig. 13 (black). The non-truncated FID and its spectrum are plotted in red for comparison. Expectedly, the result of reconstruction is strongly dependent on setting of $\lambda$. When the assumed sparsity is too low (Fig. 9a), the algorithm does not effectively reconstruct the FID: zero values in the truncated part are not changed much. Thus the “sinc” artifacts are not suppressed in the spectrum.

With an optimal level of sparsity, the algorithm does provide the reconstruction of the truncated part, but, as can be seen in Fig. 9b, the smaller peak of the spectrum is still neglected. The decay rate is not estimated quite accurately, besides, additional modulations of the FID arise. These modulations, in extreme cases, can be visible as peak-splittings in a spectrum, which has been reported by Stern et al. (2007) and Qu et al. (2015).

Finally, with a too high $\lambda$ (Fig. 13c), the reconstruction is too sparse—a peak is artificially narrowed (no decay in time domain) and its intensity is lowered.

In neither of the cases is the small peak on the right properly reconstructed.

To study a case easier for reconstruction, the same FID was truncated to 1024 measurement points (about half the full length of the signal). Again, reconstruction with IST-D was performed.

**Fig. 14:**
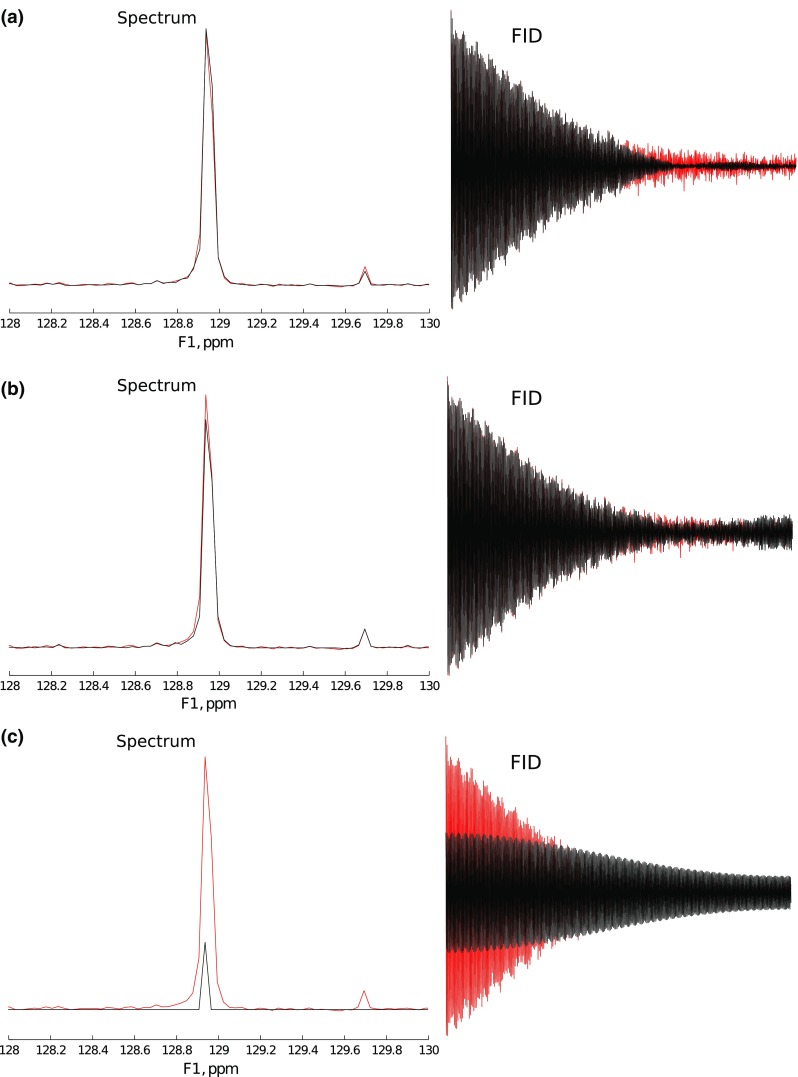
Extrapolation of 1024 points signal with IST with various thresholds: **a** Low threshold (0.2). **b** Optimal threshold (0.99). **c** High threshold (0.999)

The results are presented in Fig. 14. This time, IST algorithm works efficiently for broader range of $\lambda$—with optimum similar as before (Fig. 14b), but also very low (Fig. 14a), though, as can be seen from the FID plot, the decay rate is a little overestimated there, and also there are slight additional modulations of the FID in both cases. High sparsity (Fig. 14c) gives in this case similar results as to those of the previous case with extreme truncation: the reconstructed FID has very low decay rate.

The results confirm the observations reported before (Hyberts et al. 2012b) that 2$\times$ extrapolation using CS algorithms is rather effective.

It is interesting to note that the main difficulty of CS-based extrapolation lies in the determination of a decay rate of an FID signal. Especially for heavily truncated signals, the result depends on a proper adaptation of $\lambda$. As is known e.g. from diffusion NMR spectroscopy, the decoding of exponential decays (inverse Laplace transform) is not a trivial task (Callaghan 2011). The underestimation of decay rates leads to peak narrowing or peak splitting, while the overestimation leads to incomplete reconstruction. Perhaps some approaches to smoothen the reconstruction, similar to those known from diffusion spectroscopy (Urbańczyk et al. 2016), or others dedicated to NUS, could be useful (Hyberts et al. 2016).

### Possible modifications

Several modifications of the CS algorithms discussed above have been introduced over the years to improve their effectiveness and make them adapted to NMR spectra. Below we summarize some of the modifications.

#### Zero-filling and virtual echo

It is noteworthy that the $\ell _p$-norm used in the penalty function (2) involves both real and imaginary parts of $\varvec{x}$. Because the phase in the indirect spectral dimensions is usually known a priori, we can assume that the real part gives an absorptive Lorentzian function under FT, and the imaginary part gives a dispersive one. For decaying signals, the dispersive peaks have long “tails”, and thus ${\mathrm{Im}}(\varvec{x})$ is far from being sparse. Thus, the algorithm will strongly tend to minimize the imaginary part. This often leads to the reconstructed signal in the form of an “echo”: complex FID is combined with its own conjugated reflection. While the resulting spectrum contains a suppressed imaginary part and thus is indeed sparser, the real part is also disturbed. The common trick to avoid it is to zero-fill the signal twice at the input to the reconstruction algorithm to provide “space” for the mirror reflection (Mayzel et al. 2014). At the output, the signal is truncated back to the original size.

The observation that sparsity-constrained reconstructing algorithms tend to create an “echo” led to the invention of “Virtual Echo” concept, where the zero-filled signal is combined with its own conjugated reflection at the input (Mayzel et al. 2014). In this way, the number of the unknowns (missing points to be reconstructed) is reduced, and the effectiveness of the procedure is increased. As pointed out by Stern adn Hoch (2015), the same benefit can be achieved by changing the penalty function to use $|{\mathrm{Re}}(\varvec{x})|_{\ell _p}$ instead of $|{x}|_{\ell _p}$.

#### Automatic setting of sparsity constraint

The need to manually set up a balance $\lambda$ between the data agreement and the sparsity of the result may be considered as a difficulty in using CS methods. One of the solutions to solve it is to use the plot of the value of the first term of functional (2) vs. its second term for various settings of $\lambda$ (Hansen 1992). The curve is typically L-shaped, and experience shows that the best $\lambda$ corresponds to the point where the curve turns from a sharp decrease to a flat line. The approach might be effective, but computationally demanding, as it requires many repetitions of the reconstruction process.

Similarly costly, although with a stronger mathematical basis, is the method of Bregman iterations, where sparsity-constrained minimization is also carried out several times with different settings of $\lambda$ (Osher et al. 2005). The procedure starts from high $\lambda$, and thus in the first step only the highest components are found. Then, the signal is updated by removing these high components, and the minimization is repeated for lower $\lambda$. A somewhat simplified version of Bregman iterations, often applied in multidimensional NMR due to its robustness, is to change $\lambda$ with every iteration, starting from very high values (Hyberts et al. 2012b). Nevertheless, even using a constant value of $\lambda$ can give satisfying results (Hyberts et al. 2012b, 2014).

#### Adapting greedy methods

Greedy algorithms like CLEAN are rather ineffective in case of NMR spectra with a high dynamic range of peak intensities (Coggins et al. 2012). Improvements can be achieved by adapting the algorithm to operate on peaks rather than single points i.e. to subtract groups of points in each iteration, possibly requiring them to form a Lorentzian line. The idea was implemented in semi-automatic program by Kazimierczuk et al. (2007a) and later in other approaches (Stanek and Koźmiński 2010b; Coggins et al. 2012; Kazimierczuk and Kasprzak 2015).

#### Noise treatment

The CS signal reconstruction in NMR faces the problem of noise, which, contrary to the actual signal, is not compressible. Unfortunately, CS algorithms will anyway tend to seek for sparse, “peaky” representation of noise. To prevent them from doing so, a stopping criteria (e.g., final threshold in IST or number of peaks in OMP) or regularization parameters ($\epsilon$ in IRLS method) have to be introduced.

However, it is neither practical nor convenient to require the assumption of noise level as an input parameter. Thus, automatic settings are often desirable.

#### Remedies

The following options can be considered as remedies for CS reconstruction pitfalls:

1. Using $m \propto Klog(n/K)$ relation. Given *m* measurements of FID signal, only *K* highest spectral points are properly reconstructed with CS. Cândes et al. proposed to predict number *K* from this relation and set the $\varepsilon$ in IRLS method to be close to the *K*th highest point of the spectrum. To be more precise, it is proposed to change $\varepsilon$ parameter iteratively by putting $\varepsilon = \max \{|x^{(l)}|_{i_0}, \varepsilon _0\}$, where:
$|x |_{(i)}$ denotes the decreasing reordering of $|x_i|$-vector;
$\displaystyle i_0 = \frac{m}{4\log {n/m}}$ is the formula which is heuristically justified by $m \propto Klog(n/K)$ relation;
$x^{(l)}$ is the *l*th approximate to the solution of the IRLS problem.
  [see (Candès et al. 2008)]. One can easily imagine using a similar approach e.g. in IST, where the threshold would not be lowered more than below the *K*th highest point.
2. Keeping experimental points unperturbed. This is the approach used in IST-S. It prevents the algorithm from “over-iterating” leading to false “noise-peaks”. The sparsity of the result depends only on $\lambda$ and not on the number of iterations.
3. Checking convergence. False “sparsyfication” of the noise can be avoided by interrupting the reconstruction procedure once the change in the residual of the reconstruction is low. Usually, however, the reconstruction is carried out for each point of the direct spectral dimension separately. This may lead to peak shape disturbances along that dimension if the algorithm stops at different stages due to some local minima.
4. Cross-validation (Ward 2009). Part of the sampling points (e.g. 25 %) can be used to automatically validate the result obtained from the rest of the sampling points at different sparsity levels and to select the level that fits best. The problem with this approach is that the part of the data used for cross-validation is wasted, i.e., it does not contribute to the final spectrum. Very recently the effectiveness of cross-validation method has been demonstrated on NUS NMR data (Wu et al. 2016).
5. Bootstrap (Efron 1982). Data can be divided into subsets, and a spectrum can be reconstructed from each of them. False peaks originating from noise or reconstruction artifacts will appear at different positions, depending on a sampling schedule, while the actual resonances will stay constant. Again, the problem is the sensitivity loss due to the data division, as well as longer reconstruction times.
6. In case of methods that change experimental points, the final sparse spectrum may be corrected for possibly missing peaks and look more natural if the residual of the reconstruction is re-added to the spectrum. It should be remembered, however, that when decaying sampling density is used to improve sensitivity (Barna et al. 1987), an appropriate scaling factor for the residual has to be introduced.

## Conclusion

Sparsity-constrained reconstructions have dominated the field of non-uniform sampling in recent years. We have discussed the properties of these algorithms, in particular their basic principles and influence of crucial parameters. The above discussion is definitely not complete, and thus we encourage readers to experiment with MATLAB codes of the methods discussed included in the Supplementary Data. Our intention was to make the example codes as simple as possible. In fact, the software packages available on the market contain several additional optimizations and often automatic setup of many parameters. Still, we find it didactic to see how the algorithms work in their most generic versions. We hope that a closer look will explain some of the mysterious aspects of apparently ’black box’ techniques.

## Electronic supplementary material

Below is the link to the electronic supplementary material.

Supplementary material 1 (avi 9161 KB)

Supplementary material 2 (avi 10659 KB)

Supplementary material 3 (avi 10954 KB)

Supplementary material 4 (avi 10627 KB)

**Supporting information** MATLAB codes of the algorithms shown in the paper: OMP, IST-S, IST-D, IRLS and low-rank reconstruction. Videos showing point-by-point reconstruction of spectra from Figs. 4 and 5 (all available at: http://www.nmr.cent.uw.edu.pl, go to: Download $\rightarrow$ Compressed Sensing). (pdf 128 KB)
