# Supplementary material for: Pitfalls in compressed sensing reconstruction and how to avoid them
Source: J Biomol NMR. 2016 Nov 11;68(2):79–98. doi: 10.1007/s10858-016-0068-3 (PMC5504175; doi:10.1007/s10858-016-0068-3)
Supplement: Supplementary file 5 — Supporting information MATLAB codes of the algorithms shown in the paper: OMP, IST-S, IST-D, IRLS and low-rank reconstruction. Videos showing point-by-point reconstruction of spectra from Figs. 4 and 5 (all available at: http://www.nmr.cent.uw.edu.pl, go to: Download $\rightarrow$→ Compressed Sensing). (pdf 128 KB) [file 10858_2016_68_MOESM5_ESM.pdf]

Journal of Biomolecular NMR manuscript No.
(will be inserted by the editor)

Supplementary material for: Pitfalls in compressed sensing reconstruction and how to avoid them

Alexandra Shchukina[1,3] · Paweł Kasprzak[2] · Rupashree Dass[1] · Michał Nowakowski[1] · Krzysztof Kazimierczuk[1]

the date of receipt and acceptance should be inserted later

[1] Centre of New Technologies, University of Warsaw, Banacha 2C, 02-097 Warsaw, Poland
E-mail: k.kazimierczuk@cent.uw.edu.pl
[2] Department of Mathematical Methods in Physics, Faculty of Physics, University of Warsaw, Pasteura 5, Warsaw, Poland
[3] Institute for Spectroscopy, Russian Academy of Sciences, Fizicheskaya 5, 108840 Troitsk, Moscow, Russia

---------------OMP---------------

```
function X = omp(y, U, N, options)

% INPUT
% y - measurement vector (time domain, dimensions M x 1)
% U - sampling scheme with increment numbers (M x 1)
% N - length of full signal

% PARAMETRES
% options.max_iter - limit of iterations
% options.res ∈(0; ...) - residue norm (should be equal to noise norm)

% OUTPUT
% X (frequency domain, N x 1)

%%%%%%%%%%%%%% parameters %%%%%%%%%%%%%
res = options.res;

if isfield(options, 'max_iter')
    max_iter = options.max_iter;
else
    max_iter = 500;
end

%%%%%%%%%%%%%% initialization %%%%%%%%%%%%%
F = ifft(eye(N)); % inverse Fourier transform matrix
A = F(U, :); % measurement matrix: x = A*y
X = zeros(N, 1);
supp = [];
r = y - A*X;

%%%%%%%%%%%%%% main loop %%%%%%%%%%%%%
for i = 1:max_iter
%     disp(horzcat('iteration', ' ', int2str(i)))
    if norm(r) >= res
        x_omp = abs(A'*(y - A*X));
        [a, Index] = max(x_omp);
        supp = union(supp, Index);
        M = A(:, supp);
        X(supp, 1) = (M'*M)^(-1)*M'*y;
        r = y - A*X;
    else
        disp('stopping criterion has been reached')
        break
    end
end
end
```

---------------IST_D---------------

```
function X = ist_d(y, U, N, options)

% INPUT
% y - measurement vector (time domain, dimensions M x 1)
% U - sampling scheme with increment numbers (M x 1)
% N - length of full signal

% PARAMETRES
% options.res (0; 1) - relative to the highest peak in the spectrum with
artifacts
% options.max_iter - limit of iterations

% OUTPUT
% X (frequency domain, N x 1)
```

```
% POSSIBLE MODIFICATIONS:
% linear change of the threshold from iteration to iteration: options.change_thr
= 1

%%%%%%%%%%%% parameters %%%%%%%%%%%%
res = options.res;

if isfield(options, 'max_iter')
   max_iter = options.max_iter;
else
   max_iter = 500;
end

if isfield(options, 'threshold')
   threshold = options.threshold;
else
   threshold = 0.99;
end

%%%%%%%%%%%% modifications %%%%%%%%%%%%
if isfield(options, 'change_thr')
   change_thr = options.change_thr; % = 1
else
   change_thr = 0;
end

%%%%%%%%%%%% initialization %%%%%%%%%%%%
F = ifft(eye(N)); % inverse Fourier transform matrix
A = F(U, :); % measurement matrix: y = A*x
sp_art = A'*y; % spectrum with artifacts
t = threshold*max(abs(sp_art));
X = zeros(N, 1);
r = y;

%%%%%%%%%%%% main loop %%%%%%%%%%%%
for i = 1:max_iter
%     disp(horzcat('iteration', ' ', int2str(i)))
   if norm(r) >= res
       X = X + delta(sp_art, t);
       r = y - A*X;
       fX = zeros(N, 1);
       fX(U) = r;
       sp_art = fft(fX);
       t = threshold*max(abs(sp_art));
       if change_thr == 1 % modification 1
           t = t*threshold*(max_iter - i)/max_iter;
       end
   else
       disp('stopping criterion has been reached')
       break
   end
end
end
```

---------------IST_S---------------

```
function X = ist_s(y, U, N, options)

% INPUT
% y - measurement vector (time domain, dimensions M x 1)
% U - sampling scheme with increment numbers (M x 1)
% N - length of full signal

% PARAMETRES
% options.threshold ∈(0; 1) - relative to the highest peak in the spectrum with
```

```
artifacts
% options.max_iter - limit of iterations

% OUTPUT
% X (frequency domain, N x 1)

%%%%%%%%%%%% parameters %%%%%%%%%%%%
if isfield(options, 'max_iter')
    max_iter = options.max_iter;
else
    max_iter = 500;
end

if isfield(options, 'threshold')
    threshold = options.threshold;
else
    threshold = 0.99;
end

%%%%%%%%%%%% initialization %%%%%%%%%%%%
y0 = zeros(N, 1);
y0(U) = y;
F = ifft(eye(N)); % inverse Fourier transform matrix
A = F(U, :); % measurement matrix: y = A*x
sp_art = A'*y; % spectrum with artifacts
t = threshold*max(abs(sp_art));
X = zeros(N, 1);
l_last = 0;
Z = setdiff(1:N, U);

%%%%%%%%%%%% main loop %%%%%%%%%%%%
for i = 1:max_iter
%     disp(horzcat('iteration', ' ', int2str(i)))
    X = delta(sp_art, t);
    fX = F*X;
    y0(Z) = fX(Z);
    sp_art = fft(y0);
    t = threshold*max(abs(sp_art));
    t = t*threshold*(max_iter - i)/max_iter;
end
X = sp_art;
end
```

---------------Delta for IST---------------

```
function y_thresholded = delta(y, threshold)
s = abs(y) - threshold;
s = (s + abs(s))/2;
y_thresholded = sign(y).*s;
end
```

---------------IRLS---------------

```
function X = irls(y, U, N, options)

% INPUT
% y - measurement vector (time domain, dimensions M x 1)
% U - sampling scheme with increment numbers (M x 1)
% N - length of full signal

% PARAMETRES
% options.eps ∈(0; ...)
% options.max_iter - stopping criteria by number of iterations [default max_iter
= 20]
% options.norm - final p-norm set in the last iteration [default norm = 0]
% options.lambda - lambda [default = 1]
```

```
% POSSIBLE MODIFICATIONS:
% change of the norm from iteration to iteration: options.iterative = 1
[default]

% OUTPUT
% X (frequency domain, N x 1)

%%%%%%%%%%%% parameters %%%%%%%%%%%%
if isfield(options, 'eps')
    eps = options.eps;
    set_eps_flag=0;
else
    set_eps_flag=1 ;
end

if isfield(options, 'max_iter')
    max_iter = options.max_iter;
else
    max_iter = 20;
end

if isfield(options, 'norm')
    norm = options.norm;
else
    norm = 1;
end

if isfield(options, 'iterative')
    iterative = options.iterative;
else
    iterative = 0;
end

if isfield(options, 'lambda')
    lambda = options.lambda;
else
    lambda = 1;
end

%%%%%%%%%%%% initialization %%%%%%%%%%%%
k = 0;
F = ifft(eye(N)); % inverse Fourier transform matrix
A = F(U, :); % measurement matrix: x = A*y
fn = size(A, 2);
L = eye(size(A, 1));
K = size(A, 1);
X = A'*y;

if (set_eps_flag) % set regularization automatically
    x_sorted = sort(abs(X), 'descend');
    eps = x_sorted(1)/10000.;
    u = floor(K/4/log(fn/K)) + 1;
end

%%%%%%%%%%%% main loop %%%%%%%%%%%%
while k < max_iter
%     disp(horzcat('iteration', ' ', int2str(k)))
    if (set_eps_flag)
        x_sorted = sort(abs(X), 'descend');
        eps = min(eps, abs(x_sorted(u)));
    end
    if iterative
        tau = (k + 1)/max_iter*(1. - norm);
```

```
    else
        tau = 1. - norm;
    end
    W = diag(abs(X).^(1. + tau) + eps^(1. + tau));
    G2 = A*W;
    B = G2*A' + L*lambda;
    % Here, B is inverted by cholesky factorization. Works fast, but only for A
(complex, symmetric).
    B(logical(eye(size(B)))) = real(diag(B));
    R = chol(B);
    F = R\(R'\y);
    X = G2'*F;
    k = k + 1;
end
end
```

---------------Low Rank---------------

```
function X = low_rank(y, U, N, options)

% INPUT
% y - measurement vector (time domain, dimensions M x 1)
% U - sampling scheme with increment numbers (M x 1)
% N - length of full signal

% PARAMETRES
% options.lambda - data agreement/sparsity [default lambda = 10]
% options.Q - number of columns in Hankel matrix [default Q = ceil(0.1*N)]
% options.beta [default beta = 1]
% options.tau - "step" [default tau = beta = 1]
% options.tol - tolerance of convergence [default tol = 1e-8]
% options.max_iter - limit of iterations [default max_iter = Inf, no limit]

% OUTPUT
% X (frequency domain, N x 1)

% POSSIBLE MODIFICATIONS:
% interruption at low singular values: options.sv

%%%%%%%%%%%% default parameters %%%%%%%%%%%%
if isfield(options, 'max_iter')
    max_iter = options.max_iter;
else
    max_iter = Inf; % no upper limit to the number of iterations by default
end

if isfield(options, 'tol')
    tol = options.tol;
else
    tol = 1e-5;
end

if isfield(options, 'beta')
    beta = options.beta;
else
    beta = 1;
end

if isfield(options, 'tau')
    tau = options.tau;
else
    tau = beta;
end
```

```
if isfield(options, 'Q')
    Q = options.Q;
else
    Q = ceil(0.1*N);
end

if isfield(options, 'lambda')
    lambda = options.lambda;
else
    lambda = 1e3;
end

%%%%%%%%%%%%% modifications %%%%%%%%%%%%%
if isfield(options, 'sv') % modification 2
    sv = options.sv;
end

%%%%%%%%%%%%% initialization %%%%%%%%%%%%%
maxsig = abs(max(abs(y)));
y = y/maxsig;

% form Hankel operator R: R*X = H (H - Hankel matrix):
row = 1:((N - Q + 1)*Q);
row_block = floor(row/(N - Q + 1) - 1e-5); % 1e-5 is introduced to "shift"
division "backwards"
sr = ones((N - Q + 1)*Q, 1);
R = sparse(row, row - (N - Q + 1)*row_block + row_block, sr); % Hankel operator
R

% form sampling matrix Um (M x N) from sampling vector U (M x 1):
su = ones(length(U), 1);
Um = sparse([1:length(U)], U, su, length(U), N);

X = Um'*y;
x_last = X;
delta_x = 2*tol;
Z = R*X;
D = ones(Q*(N-Q+1), 1);% lagrangian multiplier

%%%%%%%%%%%%% main loop %%%%%%%%%%%%%
a = lambda*Um'*y;
b = beta*R';
c  = (lambda*Um'*Um + beta*R'*R)^(-1);

k = 0;
while delta_x >= tol && k < max_iter
%     disp(horzcat('iteration', ' ', int2str(k)))
    X = c*(a + b*(Z - D/beta));
    k = k + 1;
    A = R*X + D/beta;
    A = reshape(A, N - Q + 1, Q);
    [u, s, v] = svd(A, 'econ');
    if isfield(options, 'sv')
        if max(diag(s)) < sv/beta
            x = zeros(N, 1);
            break
            'singular value limit has been reached'
        end
    end
    Z = u*max(0, s - 1/beta)*v'; % soft thresholding on sihgular values
    Z = reshape(Z, Q*(N - Q + 1), 1);
    D = D + tau*(R*X - Z);
    delta_x = norm(x_last - X)/norm(x_last);
    x_last = X;
```

```
end
X = X*maxsig;
end
```
